# Associations of human milk oligosaccharides and bioactive proteins with infant growth and development among Malawian mother-infant dyads

**DOI:** 10.1093/ajcn/nqaa272

**Published:** 2020-10-23

**Authors:** Josh M Jorgensen, Rebecca Young, Per Ashorn, Ulla Ashorn, David Chaima, Jasmine C C Davis, Elisha Goonatilleke, Chiza Kumwenda, Carlito B Lebrilla, Kenneth Maleta, Elizabeth L Prado, John Sadalaki, Sarah M Totten, Lauren D Wu, Angela M Zivkovic, Kathryn G Dewey

**Affiliations:** Department of Nutrition, University of California, Davis, CA, USA; Department of Nutrition, University of California, Davis, CA, USA; Faculty of Medicine and Life Sciences, Centre for Child Health Research, University of Tampere, Tampere, Finland; Department of Pediatrics, Tampere University Hospital, Tampere, Finland; Faculty of Medicine and Life Sciences, Centre for Child Health Research, University of Tampere, Tampere, Finland; Department of Community Health, University of Malawi College of Medicine, Blantyre, Malawi; Department of Chemistry, University of California, Davis, CA, USA; Department of Chemistry, University of California, Davis, CA, USA; Department of Community Health, University of Malawi College of Medicine, Blantyre, Malawi; School of Agricultural Sciences, Department of Food Science and Nutrition, University of Zambia, Lusaka, Zambia; Department of Chemistry, University of California, Davis, CA, USA; Department of Biochemistry and Molecular Medicine, University of California, Davis, CA, USA; Department of Community Health, University of Malawi College of Medicine, Blantyre, Malawi; Department of Nutrition, University of California, Davis, CA, USA; Department of Community Health, University of Malawi College of Medicine, Blantyre, Malawi; Department of Chemistry, University of California, Davis, CA, USA; Department of Chemistry, University of California, Davis, CA, USA; Department of Nutrition, University of California, Davis, CA, USA; Foods for Health Institute, University of California, Davis, CA, USA; Department of Nutrition, University of California, Davis, CA, USA

**Keywords:** human milk oligosaccharides, bioactive breast milk proteins, infant growth, infant motor development, infant cognitive development

## Abstract

**Background:**

Human milk oligosaccharides (HMOs) and bioactive breast milk proteins have many beneficial properties. Information is sparse regarding associations between these milk constituents and infant growth and development in lower-income countries.

**Objectives:**

We aimed to examine associations of milk content of HMOs and bioactive proteins at 6 mo postpartum with infant growth and motor and cognitive development. These are secondary analyses of a randomized controlled trial in rural Malawi.

**Methods:**

Breast milk samples were analyzed at 6 mo (*n* = 659) for general categories of HMOs (total HMOs, fucosylated HMOs, and sialylated HMOs), 51 individual HMOs, and 6 bioactive proteins (lactalbumin, lactoferrin, lysozyme, antitrypsin, IgA, and osteopontin). We examined associations of the relative abundances of HMOs and concentrations of bioactive proteins with infant growth from 6 to 12 mo [change in length-for-age (ΔLAZ), weight-for-age, weight-for-length, and head circumference *z*-scores] as well as ability to stand or walk alone at 12 mo, and motor and language skills, socioemotional development, executive function, and working memory at 18 mo. Analyses were adjusted for covariates and multiple hypothesis testing.

**Results:**

Among all participants, there were inverse associations of IgA and lactoferrin concentrations with motor skills (*P* = 0.018 and *P* = 0.044), and a positive association of lactalbumin concentration with motor skills (*P* = 0.038). Among secretors only [fucosyltransferase 2 gene (*FUT2*) positive], there were positive associations of absolute abundance of HMOs with ΔLAZ (*P* = 0.035), and relative abundance of fucosylated and sialylated HMOs with language at 18 mo (*P* < 0.001 and *P* = 0.033, respectively), and inverse associations of osteopontin with standing and walking at 12 mo (*P* = 0.007 and 0.002, respectively). Relative abundances of several individual HMOs were associated with growth and development, mostly among secretors.

**Conclusions:**

Certain bioactive breast milk proteins and HMOs are associated with infant growth and motor and cognitive development. Further studies are needed to determine if a causal relation exists.

This trial was registered at clinicaltrials.gov as NCT01239693.

## Introduction

The WHO recommends exclusive breastfeeding for the first 6 mo of life (1). Breast milk is a rich source of many nutrients and other constituents that are beneficial to infants, including human milk oligosaccharides (HMOs) and bioactive breast milk proteins. HMOs provide multiple health benefits to infants by inhibiting pathogen attachment to epithelial cells in the small intestine (2), acting as prebiotics to influence beneficial changes to the composition and/or activity of gastrointestinal microbes (3), modulating intestinal immune responses (3, 4), and potentially enhancing neurodevelopment (5). Bioactive proteins exhibit bacteriostatic or bactericidal properties (6, 7), while encouraging the growth of beneficial bacteria (8, 9), function as antiadhesive antimicrobials (10), modulate inflammatory and immune responses (8, 11), aid the survival of other bioactive proteins in the infant gastrointestinal tract (12), and have been shown to enhance immunological (13), intestinal (14), and neurological and cognitive development (15).

The potential reduction in intestinal infection and modulation of the inflammatory response stimulated by HMOs and bioactive proteins might contribute to improved growth and development by allowing the infant to absorb and utilize nutrients more efficiently (16). However, there is limited evidence of associations of HMOs with infant growth and development in lower-income countries, and we could find no evidence of associations of bioactive proteins with infant growth and development. Studies in Finland (17), Denmark (18), and the United States (19) demonstrated that 2′-fucosyllactose (2′FL) was positively associated and lacto-*N*-neotetraose (LNnT) and lacto-*N*-fucopentaose I (LNFP I) were inversely associated with child height and weight, whereas 2′FL at 1 mo was positively associated with infant cognitive development at 24 mo among infants in the United States (20). The authors of those articles speculate that the HMOs affect infant growth via modulation of the infants’ intestinal microbiota. However, the composition of intestinal microbiota of infants in high-income countries has been shown to be different than that in lower-income countries (21–23), due not only to different environmental exposures, but also to differing diets (22, 23). It is not clear whether those same or alternate HMOs are associated with growth among infants in lower-income countries. To our knowledge, there has been no large-scale longitudinal study to examine associations between HMOs or bioactive breast milk proteins and infant growth and development in a lower-income country.

We examined whether HMOs and selected bioactive proteins in the milk of Malawian women at 6 mo postpartum were associated with infant growth from 6 to 12 mo, or with motor or cognitive development of their infants at 12 and 18 mo.

## Methods

The International Lipid-Based Nutrient Supplements (iLiNS) Project DYAD-Malawi trial was a randomized, controlled, outcome assessor–blinded supplementation trial of mother-child dyads in a partly semiurban, partly rural area of the Mangochi District in Malawi. The study has been described in detail elsewhere (24). Briefly, pregnant women of ≤20 wk gestation who met inclusion criteria were randomly assigned to 1 of 3 groups. Women either consumed: *1*) iron and folic acid during pregnancy; *2*) a multiple micronutrient capsule during pregnancy and up to 6 mo postpartum; or *3*) a lipid-based nutrient supplement (LNS) during pregnancy and up to 6 mo postpartum. Infants of women in the LNS group consumed from 6 to 18 mo of age an infant formulation of LNS. Infants in all 3 groups were followed up to 18 mo of age.

At enrollment, trained study staff measured maternal weight and height (from which BMI was calculated), and recorded information about parity, education, and socioeconomic status (household assets and food insecurity). Weight and height were measured in triplicate using high-quality scales (SECA 874 flat scale; Seca GmbH & Co) and stadiometers (Harpenden stadiometer; Holtain Ltd). Household asset *z*-score (HHAZ) includes information on building materials of the house, sources of water, type of lighting used in the house, type of cooking fuel used in the house, and sanitary facility. HHAZ and food security indices were created as previously described (25, 26). At the enrollment visit, trained study nurses drew blood to analyze hemoglobin (Hb) and HIV status. Hb was measured using the Hemocue 201+ system (Hemocue). HIV was analyzed using a whole-blood antibody rapid test (Alere Determine HIV-1/2; Abbott).

At 6 mo postpartum, mothers were asked to manually express the full content of 1 breast into a sterile plastic cup. Study staff thoroughly mixed the contents with a spoon, and then transferred 10 mL to storage cryovials, which were stored at −80°C until analysis. HMOs were analyzed by a nano-LC-chip/time-of-flight MS method described previously (27). To minimize batch effects, a quality control set of standards was employed to calibrate and normalize all the samples. We report total HMO and osteopontin as normalized ion counts, meaning that for each batch, the ion counts were adjusted to the quality control sample so that variations in signals between the different runs can be adjusted for. Lactoferrin, lactalbumin, lysozyme, antitrypsin, IgA, and osteopontin were analyzed by multiple reaction monitoring as described previously (28) and all but osteopontin are reported as grams per liter. Standards were not available for the HMOs at the time of analysis, so results are reported as absolute abundance (ion counts) or relative abundance (proportion of all the HMOs). We did not have a measurement of the quantity of breast milk consumed, so we deemed relative abundance as a better indicator of the consumption of individual HMOs by the infant.

Study staff measured length, weight, and head circumference of infants with a high-quality length board (Harpenden Infantometer; Holtain Ltd), infant weighing scale (SECA 381 baby scale; Seca GmbH & Co), and nonstretchable measuring tape (ShorrTape; Weigh and Measure, LLC), respectively, as described previously (29). Length-for-age *z*-score (LAZ), weight-for-age *z*-score (WAZ), weight-for-length *z*-score (WLZ), and head circumference *z*-score (HCZ) were all calculated using the WHO growth standards (30).

Methods for analyzing infant development have been described elsewhere (31). At 12 and 18 mo, infants visited their local hospital or clinic. At 12 mo, project staff observed whether the child was able to stand or walk alone. At 18 mo motor development was assessed by the Kilifi Development Inventory. The child's score was the total number of skills he or she was able to perform out of 34 fine motor skills and 35 gross motor skills. Language development score was the total number of words the child was able to say out of a 100-word vocabulary checklist based on the MacArthur–Bates Communicative Development Inventory. Socioemotional development was assessed by the Profile of Social and Emotional Development, which measures social cognition, independence, emotional lability, maladaptive behavior, and social competence. A higher score indicates greater social-emotional competence. We assessed working memory and executive function by a version of the A-not-B task, in which the child was required to remember during a delay of 5 s whether a snack was hidden under a cup on the right or left side of a board. The score was the total correct of 10 trials. Data collectors rated the child's mood, activity level, and willingness to interact during assessment, and administered the Family Care Indicators interview, which measures nurturing care and learning opportunities in the home environment.

The outcomes we analyzed are listed in **Table 1**. The bioactive proteins and groups of HMOs examined as predictor variables are listed in **Table 2**, whereas the names and specific components of the individual HMOs are listed in **Table 3**. We prespecified as the primary analyses the models for the associations of outcomes with all 6 proteins and 3 general categories of HMOs [absolute abundance of all HMOs (total HMOs), and relative abundance of fucosylated HMOs, and sialylated HMOs]. We expected the bioactive proteins and groups of HMOs to be positively associated with growth. We considered as exploratory analyses the associations of growth and development with 2 groups of HMOs [those that were both fucosylated and sialylated, and nonfucosylated neutral (undecorated) HMOs] and each of the individual HMOs.

**TABLE 1:** Outcome variables for the analyses of associations of HMOs and bioactive proteins with growth and development^1^

Outcome	*n* (%)^2^	Mean ± SD or % or median (25th, 75th percentile)
ΔLAZ from 6 to 12 mo	519 (80)	−0.27 ± 0.70
ΔWAZ from 6 to 12 mo	523 (81)	−0.23 ± 0.60
ΔWLZ from 6 to 12 mo	518 (80)	−0.41 ± 0.82
ΔHCZ from 6 to 12 mo	522 (81)	−0.41 ± 0.58
Standing at 12 mo	563 (87)	84%
Walking at 12 mo	564 (87)	54%
Motor skills at 18 mo	614 (95)	39 (36, 41)
Language (words spoken) at 18 mo	618 (96)	25 (11, 47)
Social emotional development at 18 mo	618 (96)	22 (18, 26)
Working memory and executive function at 18 mo	519 (80)	7 (4, 8)

1 HMO, human milk oligosaccharide; ΔHCZ, change in head circumference *z*-score; ΔLAZ, change in length-for-age *z*-score; ΔWAZ, change in weight-for-age *z*-score; ΔWLZ, change in weight-for-length *z*-score.

2 Percentage of infants from whom maternal HMO abundance was available (*n* = 647).

**TABLE 2:** Concentrations of bioactive proteins and abundances of groups of HMOs among secretors and nonsecretors^1^

Variable	Description	Concentration or abundance among secretors (*n* = 485)	Concentration or abundance among nonsecretors (*n* = 162)
Antitrypsin	Protein, g/L	0.035 (0.026, 0.044)	0.034 (0.026, 0.044)
IgA^2^	Protein, g/L	0.32 (0.25, 0.39)	0.33 (0.26, 0.43)
Lactalbumin^3^	Protein, g/L	1.30 (1.18, 1.50)	1.34 (1.19, 1.49)
Lactoferrin^4^	Protein, g/L	0.98 (0.75, 1.25)	0.95 (0.71, 1.16)
Lysozyme	Protein, g/L	0.05 (0.03, 0.07)	0.05 (0.03, 0.08)
Osteopontin^5^	Protein (ion counts)	73,555 (18,047, 102,778)	69,377 (21,147, 103,241)
Total HMO*	Absolute abundance of all HMOs (ion counts)	0.70 (0.58, 0.83)	0.57 (0.46, 0.67)
Fucosylated HMO*	Relative abundance of fucosylated HMOs, %	64 (59, 68)	57 (44, 63)
Sialylated HMO*	Relative abundance of sialylated HMOs, %	11 (9, 13)	15 (13, 17)
Fucosylated and sialylated HMO*	Relative abundance of HMOs that are both fucosylated and sialylated, %	3.5 (2.2, 4.8)	4.8 (3.3, 6.8)
Undecorated HMO*	Relative abundance of nonfucosylated neutral (undecorated) HMOs, %	28 (24, 34)	34 (27, 45)

1 Values are given as median (IQR). The bioactive proteins, as well as groups of total HMOs, fucosylated HMOs, and sialylated HMOs were considered primary analyses. Analyses among the groups of HMOs that were both fucosylated and sialylated and the nonfucosylated neutral (undecorated) HMOs were considered exploratory. *Median values differed between secretors and nonsecretors. HMO, human milk oligosaccharide.

2 There was a significant interaction between IgA and secretor status for standing at 12 mo.

3 There was a significant interaction between lactalbumin and secretor status for walking at 12 mo.

4 There was a significant interaction between lactoferrin and secretor status for working memory and executive function at 18 mo.

5 There were significant interactions between osteopontin and secretor status for standing and walking at 12 mo, and working memory and executive function at 18 mo.

**TABLE 3:** Names, compositions, and relative abundance of the HMOs analyzed^1^

Abbreviation	Composition^2^	Name	Relative abundance among secretors (*n* = 485)	Relative abundance among nonsecretors (*n* = 162)
3′SL*	2001	3′-Sialyllactose	2.1 (1.6, 2.9)	3.1 (2.1, 4.0)
6′SL*	2001	6′-Sialyllactose	0.09 (0.03, 0.16)	0.21 (0.09, 0.50)
3′FL*	2010	3-Fucosyllactose	0.22 (0.09, 0.47)	0.60 (0.18, 1.07)
2′FL*	2010	2′-Fucosyllactose	14 (10, 18)	0.32 (0.18, 0.51)
LDFT*	2020	Lactodifucotetraose	4.2 (1.0, 8.1)	0.08 (0.05, 0.15)
LNT*	3100	Lacto-*N*-tetraose	14 (11, 17)	22 (16, 30)
LNnT*	3100	Lacto-*N*-neotetraose	8.5 (6.5, 10.5)	6.8 (3.9, 9.4)
LNT + LNnT*	3100	Lacto-*N*-tetraose + lacto-*N*-neotetraose	23 (19, 27)	29 (23, 37)
LSTa*	3101	Sialyllacto-*N*-neotetraose (a)	0.35 (0.22, 0.51)	0.45 (0.27, 0.73)
LSTb*	3101	Sialyllacto-*N*-tetraose (b)	2.0 (1.5, 2.4)	3.3 (2.7, 3.8)
LSTc*	3101	Siallylacto-*N*-tetraose (c)	1.7 (1.3, 2.2)	1.8 (1.3, 2.4)
LNFP I + III*	3110	Lacto-*N*-fucopentaose I + III	5.6 (3.7, 9.6)	3.6 (2.2, 4.4)
LNFP II*	3110	Lacto-*N*-fucopentaose II	4.1 (0.8, 6.1)	9.6 (0.9, 11.3)
F-LSTc*	3111	Monofucosylmonosialyllacto-*N*-neotetraose	0.18 (0.09, 0.35)	0.15 (0.08, 0.56)
LNDFH + 3120*	3120	Lacto-*N*-difucohexaose I + II + unknown 3120	2.2 (0.3, 3.3)	3.4 (0.5, 4.3)
4100a	4100	No literature name	0.12 (0.08, 0.23)	0.15 (0.09, 0.26)
4100b*	4100	No literature name	0.09 (0.06, 0.12)	0.12 (0.09, 0.18)
LNH*	4200	Lacto-*N*-hexaose	0.72 (0.39, 1.24)	0.65 (0.31, 1.9)
LNnH*	4200	Lacto-*N*-neohexaose	1.7 (1.0, 2.5)	1.1 (0.4, 2.0)
p-LNH^3^	4200	*para*-lacto-*N*-hexaose	0.38 (0.19, 0.81)	0.23 (0.10, 0.45)
S-LNH*	4201	Monosialyllacto-*N*-hexaose	0.11 (0.05, 0.16)	0.16 (0.10, 0.31)
4021a + S-LNnH II*	4201	No literature name + sialyllacto-*N*-neohexaose II	0.56 (0.33, 0.89)	0.35 (0.16, 0.70)
MFpLNH IV*	4210	Fucosyl-*para*-lacto-*N*-hexaose	2.8 (2.2, 3.4)	3.3 (2.4, 3.9)
4120a*	4210	No literature name	0.08 (0.04, 0.27)	0.14 (0.05, 0.57)
MFLNH I + III*	4210	Monofucosyllacto-*N*-hexaose I + III	2.5 (1.6, 3.3)	3.3 (2.2, 4.9)
IFLNH III*	4210	Isomer 3 fucosyl-*para*-lacto-*N*-hexaose	1.8 (1.2, 2.2)	1.5 (0.7, 2.3)
IFLNH I*	4210	Isomer 1 fucosyl-*para*-lacto-*N*-hexaose	0.24 (0.07, 0.75)	0.06 (0.03, 0.16)
4211a	4211	No literature name	0.21 (0.07, 0.32)	0.35 (0.12, 0.40)
4211b*	4211	No literature name	0.12 (0.07, 0.19)	0.29 (0.15, 0.31)
4211c*	4211	No literature name	1.9 (1.5, 2.3)	2.4 (1.9, 3.0)
DFLNHa*	4220	Difucosyllacto-*N*-hexaose (a)	0.82 (0.36, 1.84)	0.10 (0.05, 0.15)
DFLNHb*	4220	Difucosyllacto-*N*-hexaose (b)	1.33 (0.83, 2.01)	4.3 (3.0, 5.7)
DFLNHc*	4220	Difucosyllacto-*N*-hexaose (c)	0.11 (0.05, 0.21)	0.06 (0.03, 0.19)
DFpLNH II*	4220	Difucosyl-*para*-lacto-*N*-hexaose	1.9 (1.4, 2.3)	2.2 (1.6, 2.9)
DFS-LNnH*	4221	Difucosylmonosialyllacto-*N*-neohexaose	0.03 (0.04, 0.13)	0.03 (0.04, 0.05)
TFLNH*	4230	Trifucosyllacto-*N*-hexaose	1.01 (0.11, 1.37)	0.7 (0.1, 1.0)
4240a*	4240	No literature name	0.11 (0.04, 0.22)	0.03 (0.02, 0.04)
4320a*	4320	No literature name	0.10 (0.05, 0.19)	0.08 (0.04, 0.18)
5130a*	5130	No literature name	0.34 (0.15, 0.61)	0.45 (0.24, 0.89)
5130b*	5130	No literature name	0.09 (0.05, 0.19)	0.11 (0.06, 0.18)
5130c*	5130	No literature name	0.10 (0.04, 0.27)	0.07 (0.02, 0.21)
5230a + DFLNnO I/DFLNO II*	5230	Difucosyllacto-*N*-neooctaose I/difucosyllacto-*N*-octaose II + 5230	0.76 (0.45, 0.98)	0.56 (0.23, 1.04)
5230a	5230	No literature name	0.17 (0.04, 0.34)	0.18 (0.04, 0.40)
5230b	5230	No literature name	0.14 (0.08, 0.22)	0.17 (0.10, 0.29)
5300a^4^	5300	No literature name	0.47 (0.20, 0.78)	0.30 (0.14, 0.65)
F-LNO*	5310	Fucosyllacto-*N*-octaose	0.54 (0.33, 0.74)	0.54 (0.31, 0.89)
5311a	5311	No literature name	0.05 (0.03, 0.09)	0.05 (0.03, 0.10)
DFLNO I*	5320	Difucosyllacto-*N*-octaose I	0.31 (0.15, 0.62)	0.84 (0.26, 1.30)
DFLNnO II*	5320	Difucosyllacto-*N*-neooctaose II	0.23 (0.10, 0.44)	0.34 (0.08, 0.77)
DFLNnO I/DFLNO II*	5320	Difucosyllacto-*N*-neooctaose I/difucosyllacto-*N*-octaose II	0.44 (0.09, 0.79)	0.13 (0.05, 0.53)
5330a	5330	No literature name	0.04 (0.03, 0.15)	0.05 (0.03, 0.06)
6400a^5^	6400	No literature name	0.03 (0.02, 0.06)	0.03 (0.02, 0.05)
6400b	6400	No literature name	0.05 (0.04, 0.11)	0.05 (0.03, 0.07)

1 Values for abundance are given as median (IQR). *Median values differ between secretors and nonsecretors. HMO, human milk oligosaccharide.

2 Composition given as hexose, *N*-acetylhexoseamine (HexNAc), fucose, *N*-acetylneuraminic acid (sialic acid). For example, 5311 has 5 hexoses, 3 HexNAc, 1 fucose, and 1 sialic acid. Thus, those with a nonzero number in the last position are sialylated; those with a nonzero number in the third position are fucosylated.

3 There was a significant interaction between p-LNH and secretor status for language skills at 18 mo.

4 There was a significant interaction between the unnamed HMO 5300a and secretor status for language skills at 18 mo.

5 There was a significant interaction between the unnamed HMO 6400a and secretor status for walking at 12 mo.

Spearman correlation coefficients were used to assess associations of the bioactive proteins with groups of HMOs and individual HMOs. Covariate adjusted regression models were used to examine associations of abundances of the 5 groups of HMOs (total HMOs, fucosylated HMOs, sialylated HMOs, fucosylated and sialylated HMOs, and undecorated HMOs) and bioactive proteins in all women with the infant growth and development outcomes. The method of determining secretor status was described previously (32). Women with >6% relative abundance of known α1–2-linked fucosylated HMOs [2′FL, lactodifucotetraose (LDFT), trifucosyllacto-*N*-hexaose, difucosyllacto-*N*-hexaose a (DFLNHa), difucosyllacto-*N*-hexaose c (DFLNHc), and fucosyl-paralacto-*N*-hexaose-I (IFLNH I)] were considered phenotypic secretors. Women with the secretor phenotype are fucosyltransferase 2 gene (*FUT2*) positive and produce breast milk with a much higher proportion of α1–2-linked fucosylated HMOs compared with women with the nonsecretor phenotype. Using the nonparametric Kruskal–Wallis test, we examined whether abundances of the groups of HMOs and individual HMOs, and concentrations of proteins differed between women with the secretor phenotype and nonsecretors. Covariate adjusted regression models were examined separately in secretors and in nonsecretors for those HMOs that differed between secretors and nonsecretors, and also when the interaction between secretor status and the predictor variable was significant. Otherwise, secretors and nonsecretors were analyzed together. Covariate-adjusted linear regression was performed to assess associations between HMOs/proteins and continuous outcomes, and logistic regression was used for dichotomous outcomes after controlling for preselected covariates. For the primary analyses, when there were significant associations we explored differences in outcomes between infants of mothers with HMO or protein content below compared with above the median value by using generalized linear models.

Covariates included in all models were baseline maternal age, height, BMI, parity, education, food security, HIV status, Hb, household assets, and residential location, as well as infant sex and season at the time of sample collection, and intervention group. Additionally, in models for the 4 development outcomes assessed at 18 mo (motor skills, vocabulary, socioemotional development, and executive function/working memory), we included a Family Care Indicators score. This score is the sum of the source of play materials (3 items), variety of play materials (7 items), whether or not books or magazines were present in the home (2 items), and activities items (6 items) indicating whether any adult has engaged in each of 6 activities in the past 3 d. Analyses of motor development/executive function and working memory were further controlled for the child's mood, activity level, and willingness to interact with the tester. For all regression models the residuals were checked for normality, and homoskedasticity was evaluated via Q-Q plots, Shapiro–Wilk testing, and Breusch–Pagan testing.

We adjusted for multiple hypothesis tests by using the Benjamini–Hochberg (BH) procedure, using a false discovery rate of 15%. Groups for the BH procedure were formed based on the outcome variable. We did not adjust for multiple hypothesis testing for the exploratory analyses.

## Results

Of the 1391 women enrolled in the study, 869 were assigned to the complete intervention group and followed up to 18 mo after delivery. Of those, we successfully collected breast milk from 659 at 6 mo postpartum, and successfully analyzed HMOs from 647 and proteins from 637 samples (**Supplemental Figure 1**). Sample sizes and average values or percentages for each of the outcomes among the infants included in the study are described in Table 1. The mean (± SD) maternal age at study enrollment was 25.0 ± 6.0 y; the mean (± SD) maternal BMI at study enrollment was 22.0 ± 2.7 kg/m^2^; the median (25th, 75th percentile) maternal years of formal education was 3 (0, 6) y; and 47.3% of infants were males.

In this population, 75% of women had the secretor phenotype. No milk proteins differed between secretors and nonsecretors (Table 2), although there were interactions between secretor status and particular proteins with regard to the relation with development outcomes (as noted in Table 2). The absolute abundance of total HMOs and the relative abundance of all groups of HMOs differed between secretors and nonsecretors (Table 2), as did the HMOs marked with asterisks in Table 3. Among the HMOs that did not differ between secretors and nonsecretors, the HMOs for which there was an interaction between secretor status and that HMO for any of the outcomes are noted in Table 3.

There were several significant associations of the bioactive breast milk proteins with the other bioactive proteins, groups of HMOs, or individual HMOs (**Supplemental Table 1**). All of the bioactive proteins were positively associated with the other proteins, except for IgA and lactalbumin. Antitrypsin concentration was positively associated with the absolute abundance of all HMOs and relative abundance of sialylated HMOs, HMOs that are both fucosylated and sialylated, and 13 individual HMOs, and negatively associated with 14 individual HMOs. IgA concentration was positively associated with sialylated HMOs, HMOs with both fucose and sialic acid, and 6 individual HMOs, and negatively associated with undecorated (nonfucosylated neutral) HMOs and 17 individual HMOs. Lactalbumin concentration was positively associated with sialylated HMOs, HMOs that were both fucosylated and sialylated, and 16 individual HMOs, and negatively associated with 19 individual HMOs. Lactoferrin concentration was positively associated with fucosylated HMOs and 13 individual HMOs, and inversely associated with 13 individual HMOs. Lysozyme concentration was positively associated with sialylated HMOs and 7 individual HMOs, and negatively associated with 8 individual HMOs. Osteopontin content was positively associated with 14 individual HMOs and inversely associated with 18 individual HMOs.

Many associations of HMOs and bioactive proteins with infant growth and development were significant, as described below. For the significant associations among the exploratory analyses (group of HMOs that are both fucosylated and sialylated, group of neutral, nonfucosylated HMOs, and individual HMOs), the outcome values at the first and fifth quintiles of the predictor HMO or group of HMOs are presented in **Supplemental Table 2**.

### Associations of milk constituents with infant growth from 6 to 12 mo (change in LAZ, WAZ, WLZ, and HCZ)

**FIGURE 1:**
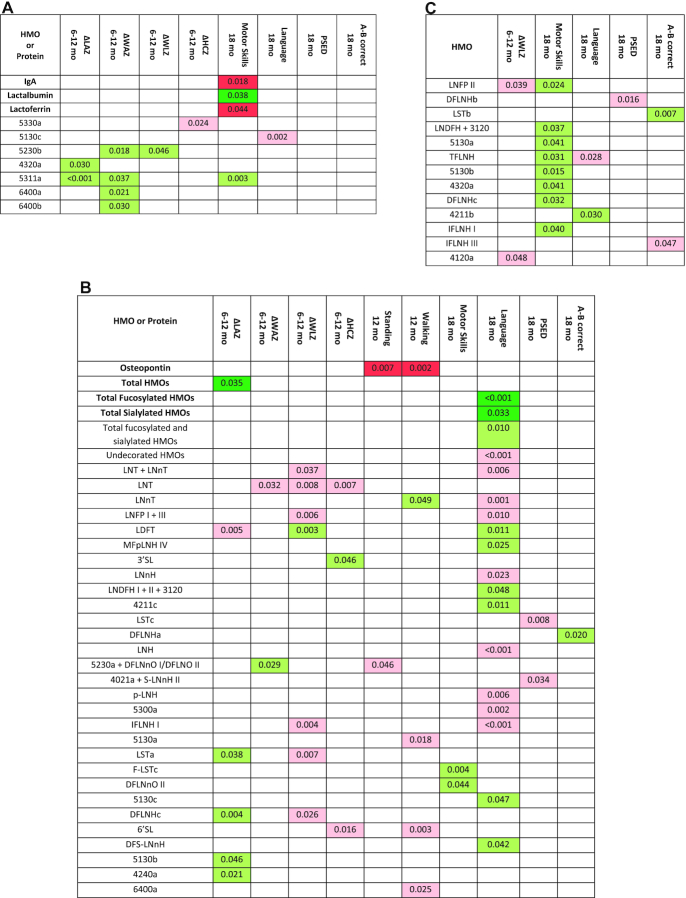
Associations of human milk oligosaccharides (HMOs) and bioactive proteins at 6 mo with infant growth and development in 659 Malawian mother-infant dyads: (A) in all participants for milk constituents that did not differ between secretors and nonsecretors; and in secretors (B) and nonsecretors (C) for milk constituents that differed between secretors and nonsecretors, or for which there was a significant interaction between the milk constituent and secretor status. Only shown are the milk constituents that were significantly associated with ≥1 of the outcomes. Bioactive proteins and groups of HMOs in bold are primary analyses; nonbolded HMOs are exploratory analyses. All numbers are *P* values determined by linear regression. All models were adjusted for baseline maternal age, height, BMI, parity, education, food security, HIV status, hemoglobin, household assets, and residential location, as well as infant sex and season at the time of sample collection. Associations in dark green are primary analyses that were in the positive direction and remained significant after the Benjamini–Hochberg correction for multiple hypothesis testing. Associations in light green are exploratory HMOs that were in the positive direction. Associations in pink are exploratory HMOs that were in the negative direction. Associations in red are primary analyses that were in the negative direction and remained significant after the Benjamini–Hochberg procedure. HMOs identified by numbers have not been previously named. Their composition is given as hexose*, N*-acetylhexosamine (HexNAc), fucose, *N*-acetylneuraminic acid (sialic acid). For example, 5311 has 5 hexoses, 3 HexNAc, 1 fucose, and 1 sialic acid. Thus, those with a nonzero number in the last position are sialylated; those with a nonzero number in the 3rd position are fucosylated. A-B correct, A-not-B working memory and executive function test; DFLNH, difucosyllacto-*N*-hexaose; DFLNnO, difucosyllacto-*N*-neooctaose; DFLNO, difucosyllacto-*N*-octaose; DFS-LNnH, difucosylmonosialyllacto-*N*-neohexaose; F-LST, fucosyl-sialyllacto-*N*-tetraose; HMO, human milk oligosaccharide; IFLNH, fucosyl-*para*-lacto-*N*-hexaose; LDFT, lactodifucotetraose; LNDFH, lacto-*N*-difucohexaose; LNFP, lacto-*N*-fucopentaose; LNH, lacto-*N*-hexaose; LNnH, lacto-*N*-neohexaose; LNnT, lacto-*N*-neotetraose; LNT, lacto-*N*-tetraose; LST, sialyllacto-*N*-tetraose; MFpLNH, fucosyl-*para*-lacto-*N*-hexaose; p-LNH, *para*-lacto-*N*-hexaose; PSED, profile of socioemotional development score; S-LNnH, sialyllacto-*N*-neohexaose; TFLNH, trifucosyllacto-*N*-hexaose; ΔHCZ, change in head circumference *z*-score; ΔLAZ, change in length-for-age *z*-score; ΔWAZ, change in weight-for-age *z*-score; ΔWLZ, change in weight-for-length *z*-score; 2′FL, 2′-fucosyllactose; 3′FL, 3′-fucosyllactose; 3′SL, 3′-sialyllactose; 6′SL, 6′-sialyllactose.

Among secretors and nonsecretors combined, for the primary analyses there were no significant associations of the abundance of groups of HMOs or concentrations of bioactive proteins with any of the 4 infant growth indicators (**Figure 1**A). For several individual HMOs, relative abundances were not different between secretors and nonsecretors, and there were no significant interactions with secretor status, therefore associations with these HMOs were examined in the combined group. Among the exploratory analyses of associations of those individual HMOs with the 4 growth indicators, 7 coefficients were positive and 1 was negative; however, the only significant association with a *P* value <0.01 was a positive association between the unnamed HMO 5311a and change in LAZ (ΔLAZ) (*P* < 0.001).

**FIGURE 2:**
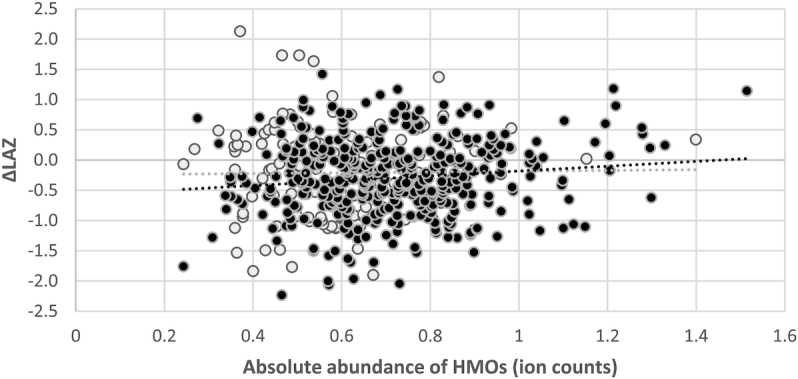
Association of change in infant length-for-age *z*-score (LAZ) from 6 to 12 mo with absolute abundance of human milk oligosaccharides (HMOs) in women with the secretor phenotype in black (*n* = 485) and nonsecretor phenotype in gray (*n* = 162). Among secretors, a one-tenth of 1 unit increase in absolute abundance of HMOs (∼15% increase in the median value) would correspond with a 0.04 (95% CI: 0.003, 0.077) unit increase in the change in LAZ from 6 to 12 mo (*P* = 0.035, assessed by covariate-adjusted linear regression). There was no significant association in nonsecretors (*P* = 0.889).

Among secretors, for the primary analyses of groups of HMOs and bioactive proteins there was 1 association that remained significant after adjusting for multiple tests (Figure 1B), a positive relation between the absolute abundance of all HMOs and ΔLAZ (*P* = 0.035; **Figure 2**). Infants of mothers with an absolute abundance of total HMOs above the median experienced, on average, 0.08 greater ΔLAZ from 6 to 12 mo than those below the median. Among the exploratory analyses of the relative abundance of individual HMOs, there were 7 positive and 11 negative associations with infant growth (*P* < 0.05). Of those, the positive associations that had a *P* value <0.01 were between LDFT and change in WLZ (ΔWLZ) (*P* = 0.003) and DFLNHc and ΔLAZ (*P* = 0.004). The inverse associations with a *P* value <0.01 were between the relative abundance of lacto-*N*-tetraose (LNT) and ΔWLZ and change in HCZ (ΔHCZ) (*P* = 0.008 and *P* = 0.007, respectively), LNFP I + III and ΔWLZ (*P* = 0.006), LDFT and ΔLAZ (*P* = 0.005), IFLNH I and ΔWLZ (*P* = 0.004), and sialyllacto-*N*-tetraose a (LSTa) and ΔWLZ (*P* = 0.007).

Among nonsecretors, there were no significant associations for the primary analyses of bioactive proteins or groups of HMOs (Figure 1C). Among the exploratory analyses, there were 2 inverse associations, neither of which had a *P* value <0.01.

### Associations of breast milk constituents with infant development

Among secretors and nonsecretors combined there were no significant associations of abundances of the groups of HMOs with infant development. Two inverse associations between the concentrations of bioactive proteins and motor skills remained significant after adjusting for multiple hypothesis testing (IgA and lactoferrin) (Figure 1A). For every 1 g/L higher value of IgA, the number of motor skills performed was 28% lower (*P* = 0.018); for every 1 g/L higher value of lactoferrin, the number of motor skills performed was 8% lower (*P* = 0.044). There was a positive association between lactalbumin concentration and motor skills that remained significant after multiple hypothesis testing. For every 1 g/L higher value of lactalbumin, the number of motor skills performed was 15% higher (*P* = 0.038). Among the exploratory analyses of associations with relative abundances of individual HMOs, there was 1 positive and 3 inverse associations with infant development. Those that had *P* values <0.01 were a positive association between the unnamed HMO 5311a and motor skills (*P* = 0.003), and an inverse association between the unnamed HMO 5130c and language at 18 mo (*P* = 0.002).

**FIGURE 3:**
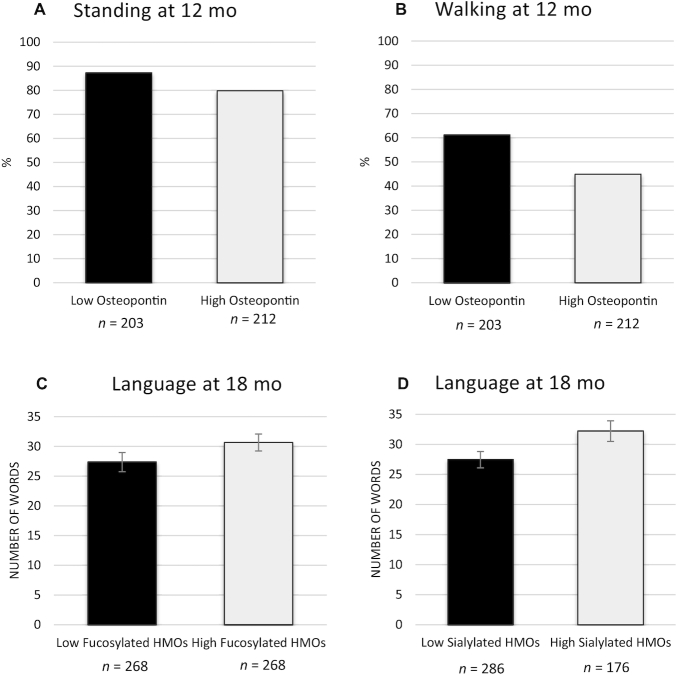
Percentage of infants standing or walking at 12 mo and mean language skills at 18 mo, in infants of secretor women with milk bioactive protein concentrations or human milk oligosaccharide (HMO) abundances below vs. above the median. Differences were assessed by generalized linear models. (A) Percentage of infants who were able to stand at 12 mo among those born to women with low vs. high milk osteopontin concentration (unadjusted *P* = 0.041; adjusted *P* = 0.021). (B) Percentage of infants who were able to walk at 12 mo among those born to women with low vs. high milk osteopontin concentration (unadjusted *P* < 0.001; adjusted *P* = 0.004). (C) Mean number of words spoken by infants at 18 mo among those born to women with low vs. high relative abundance of fucosylated HMOs (unadjusted *P* = 0.094; adjusted *P* = 0.025). (D) Mean number of words spoken by infants at 18 mo among those born to women with low vs. high relative abundance of sialylated HMOs (unadjusted *P* = 0.023; adjusted *P* = 0.026).

Among secretors (Figure 1B), for the primary analyses there were inverse associations between osteopontin and both standing and walking at 12 mo after adjusting for multiple hypothesis testing (*P* = 0.007 and *P* = 0.002, respectively). There were positive associations of both the relative abundance of fucosylated HMOs and relative abundance of sialylated HMOs with language at 18 mo, both of which remained significant after adjusting for multiple tests (*P* = 0.007 and *P* = 0.033). **Figure 3** illustrates these associations by presenting the developmental outcomes in groups of infants whose mothers had milk values above compared with below the median for each of the predictors. A greater percentage of infants of mothers with osteopontin below the median were standing at 12 mo compared with infants of mothers with osteopontin above the median (87.2% compared with 79.7%) in both unadjusted (*P* = 0.041) and adjusted (*P* = 0.021) models. Similarly, the percentage who were walking at 12 mo was significantly greater among infants of mothers with osteopontin below compared with above the median (61.1% compared with 44.8%) in both unadjusted (*P* < 0.001) and adjusted (*P* = 0.004) models. Infants of mothers with a relative abundance of fucosylated HMOs above the median had a greater vocabulary at 12 mo than infants of mothers with low fucosylated HMOs, after adjusting for covariates [median (95% CI): 22.7 (12.9 to 35.1) words compared with 18.2 (9.6 to 29.4) words; *P* = 0.025]. Similarly, infants of mothers with a high relative abundance of sialylated HMOs were able to speak more words than infants of mothers with low sialylated HMOs, after adjusting for covariates [median (95% CI): 23.9 (13.6 to 37.0) words compared with 19.3 (10.5 to 30.7) words; *P* = 0.026]. Among the exploratory analyses of relative abundances of individual HMOs, there were 11 positive and 15 inverse associations with infant development. The only positive association with a *P* value <0.01 was between fucosyl-sialyllacto-*N*-tetraose c and motor skills (*P* = 0.004), whereas 9 inverse associations had *P* values <0.01, most of which were associations with language at 18 mo [relative abundance of nonfucosylated neutral HMOs (*P* < 0.001), LNT + LNnT (*P* = 0.006), LNnT (*P* = 0.001), lacto-*N*-hexaose (*P* < 0.001), *para*-lacto-*N*-hexaose (*P* = 0.006), IFLNH I (*P* < 0.001), and the unnamed HMO 5300a (*P* = 0.002). There were also inverse associations between LSTc and social emotional development (*P* = 0.008), and 6′-sialyllactose and walking at 12 mo (*P* = 0.003).

Among nonsecretors (Figure 1C), there were no significant associations for the primary analyses. For the exploratory analyses, there were 10 positive and 3 inverse associations. The only association with a *P* value <0.01 was a positive association between the relative abundance of LSTb and working memory and executive function (*P* = 0.007).

## Discussion

In this population of Malawian mother-child dyads, the concentrations of certain bioactive breast milk proteins and abundance of groups of HMOs at 6 mo postpartum were positively associated with infant growth or development, as hypothesized, but for others the relations were nonsignificant, were in the opposite direction than expected, or differed between secretor and nonsecretor mothers. Among secretors and nonsecretors combined, milk concentration of lactalbumin was positively associated with motor skills, but concentrations of IgA and lactoferrin were inversely associated with motor skills at 18 mo. Among secretors only, the total abundance of HMOs was positively associated with ΔLAZ from 6 to 12 mo, and the total relative abundances of fucosylated HMOs and sialylated HMOs were positively associated with language development at 18 mo, whereas there were inverse associations between osteopontin concentration and the infant's ability to stand or walk alone at 12 mo. There were several significant associations of the relative abundance of individual HMOs with growth and development outcomes, particularly among women with the secretor phenotype.

We hypothesized that bioactive proteins and HMOs would be associated with improved motor and cognitive development, because the protection they afford against infection might enable enhanced physical growth and neurological development. The positive associations described above for milk lactalbumin and fucosylated or sialylated HMOs were in line with our hypotheses. However, we could find no other direct evidence from previous studies of associations between lactalbumin concentration and motor function, or between fucosylated or sialylated HMOs and language development. More studies are needed to examine these associations.

The inverse associations of IgA and lactoferrin concentrations with motor skills, and of osteopontin with standing and walking alone at 12 mo were surprising. It is possible that those bioactive proteins increased in response to an environmental trigger that also caused a delay in those particular motor skills. For example, an infant could have been exposed to a pathogen that caused an illness that inhibited motor development. The infant's mother could have been exposed to the same pathogen and responded by producing more of those particular proteins, or could have responded to signals of the infant's illness by increasing production of those particular proteins even if she was not directly exposed to the pathogen. Ultrasound evidence has shown a reverse ductal flow whereby immune secretions or pathogens in an infant's saliva (from gastrointestinal or respiratory secretions) pass back into the collecting ducts in the mammary gland to initiate a maternal response (33, 34). Such a maternal response can cause milk bioactive proteins to increase in mothers of sick infants. Breakey and colleagues (35) found an increased concentration of lactoferrin in breast milk of Argentinian mothers of sick infants. Riskin and colleagues (36) found that when the breastfeeding baby, but not mother, was sick, there was a trend toward increased lactoferrin concentration (*P* = 0.072) and significantly higher concentrations of macrophages, lymphocytes, neutrophils, and CD45^+^ cells in breast milk. In the current study population we found positive associations between the concentration of lactoferrin at 6 mo and prevalence of elevated C-reactive protein (a marker of inflammation) in infants at 18 mo (J Jorgensen, R Young, P Ashorn, U Ashorn, D Chaima, J Davis, E Goonatilleke, C Kumwenda, C Lebrilla, K Maleta, J Sadalaki, S Totten, L Wu, A Zivkovic, K Dewey, unpublished results, 2020). It is possible that the trigger that caused inflammation in the infant also caused the mother to increase production of lactoferrin. We also found elevated osteopontin among this study population during the wet season, a time when infections are more common and food is less available (J Jorgensen, R Young, P Ashorn, U Ashorn, D Chaima, J Davis, E Goonatilleke, C Kumwenda, C Lebrilla, K Maleta, J Sadalaki, S Totten, L Wu, A Zivkovic, K Dewey, unpublished results, 2020). Whether these bioactive proteins respond to infection or other deficits that inhibit motor development needs to be studied further.

Some of our results differ from what others have reported. In the same study population as ours, Charbonneau and colleagues (37) found higher total and sialylated HMOs at 6 mo postpartum in mothers of infants who were considered “healthy” (LAZ >0) compared with those who were stunted (LAZ < −2) at 6 mo of age. We did not find a positive association between sialylated HMOs and growth. One difference between studies is that Charbonneau et al. examined attained size (LAZ) at 6 mo only, whereas we examined the association with linear growth longitudinally. Furthermore, they binned children into “healthy” or “stunted,” whereas we examined the associations using continuous outcome variables. Among secretors we found a positive association between total abundance of HMOs and change in LAZ from 6 to 12 mo, whereas studies in Finland (17), Denmark (18), the United States (19), and Brazil (38) did not demonstrate such an association [among secretors only in the Finland (*n* = 699), Denmark (*n* = 30), and Brazil studies (*n* = 68), and among all participants in the US study (*n* = 25)]. We found no association of total HMOs with change in weight from 6 to 12 mo, whereas the studies in Finland (17) and Denmark (18) showed positive associations between total HMOs and weight among secretors, and the study in Brazil showed an inverse association among secretors (38). Study design could be a source of differences in findings, particularly between the current study and the Danish and Brazilian studies. In those studies, attained length was assessed cross-sectionally and weight gain was assessed retrospectively with all associations analyzed by unadjusted correlation coefficients, whereas in our study we examined change in weight and length prospectively and all associations were adjusted for covariates.

Geographical variation in infant microbiota might also explain some of the differences in findings across studies. Infants in the same catchment area as the current study had a greater proportion of beneficial bifidobacteria and lower proportions of more often harmful clostridial species, staphylococci, and *Akkermansia*-like bacteria compared with infants in Finland (21). If the composition of the intestinal microbiota is different, HMOs might interact differently with the gut bacteria in different geographical regions. In Finland, 2′FL was positively associated with change in length, but not weight, from 3 to 12 mo (17), whereas in Denmark 2′FL was positively associated with weight gain from 0 to 5 mo, but not length (18). In Los Angeles, 2′FL at 1 mo (but not at 6 mo) was positively associated with cognitive development at 24 mo in 50 mother-child dyads (20). In both Finland and Denmark, LNnT was inversely associated with length and weight (17, 18). We did not find such associations of 2′FL or LNnT with change in infant length or weight, or developmental outcomes, nor did studies in Brazil (38), The Gambia (32), and Singapore (39). The prevalence of bifidobacteria is much higher in lower-income regions of the Southern Hemisphere compared with high-income countries of the Northern Hemisphere (21–23). In our study population, bifidobacteria comprise the largest proportion of microbes in the first year of life (40) and are associated with infant growth (41). Both 2′FL and LNnT have been shown to enhance the growth of beneficial bifidobacteria (32, 42, 43). Because the proportion of bifidobacteria was already high in our study population (and likely in Brazil, The Gambia, and Singapore), consumption of additional 2′FL might not have increased bifidobacteria to the extent that it enhanced growth or development. The interactions of HMOs with infant microbiota are complex and require further research in different regions, including the association of HMOs with infant growth and development.

Among the exploratory analyses, some of our results were similar to those reported by others. Among secretors, we found a positive association between DFLNHc and linear growth, which was similar to results of small studies in Denmark and the Gambia (18, 32). We also found an inverse association between the relative abundance of LNFP I + III and ΔWLZ among secretors, similar to findings for LNFP I (by itself) in the United States (19) and Brazil (38) [note that we were unable to chromatographically separate LNFP I and LNFP III during analysis, but LNFP III is <5% the abundance of LNFP I (32)]. Finally, the inverse associations of LNT + LNnT and LSTa with change in infant WAZ were similar to associations observed in Brazil (38).

In this study population, 75% of women were defined as secretors, which is consistent with reported secretor prevalence in the same region of Africa (44, 45). We used a cutoff of <6% relative abundance of α1–2-linked fucosylated HMOs to categorize women as nonsecretors, which is the same cutoff used by Davis and colleagues (32). Others have defined nonsecretors as those who have no detectable α1–2-linked fucosylated HMOs in breast milk (45), or concentrations below a “natural, very low break in the data” (44) or “near absence” of 2′FL or LNFP I (17, 18), although exact cutoff values for the “natural break” or “near absence” were not defined. Breast milk of women categorized as nonsecretors based on blood analysis has been found to contain low concentrations of 2′FL and LDFT (46), both of which contain α1–2-linked fucoses. Therefore, defining secretors as those who contain any detectable amount of 2′FL can falsely categorize some nonsecretors as secretors, yet choosing a cutoff that is too high can falsely categorize secretors as nonsecretors. Research is needed to determine a standardized cutoff of HMO concentration to be used to accurately define secretor status.

We are aware of no other study in a low-income country that has examined the association of growth and development with the large number of HMOs and bioactive proteins that we were able to analyze. Also, a large sample size for the milk constituents gave us ample power to examine associations separately among secretors and nonsecretors, while controlling for multiple covariates. Another strength was the longitudinal study design, which allowed us to examine associations with growth over time. Concentrations of HMOs and bioactive breast milk proteins are known to change throughout the stage of lactation (32, 47, 48); however, we analyzed data from women from whom we collected breast milk within 2 wk of the scheduled 6-mo collection time point, thereby avoiding the potentially confounding influence of lactation stage. We were limited by not having standards to measure the concentrations of HMOs and osteopontin. The samples were analyzed in 2015 when few standards were available, and for the few that were available, the cost to create standard pools would have been prohibitive, which would have limited the number of HMOs assessed. Furthermore, the intent of this study was to examine associations of relative abundances of HMOs with infant outcomes, rather than claim anything truly quantitative regarding concentrations of these HMOs. We believe that use of relative abundances based on ion abundances is sufficient for this purpose. This approach allowed us to analyze associations of all 51 HMOs with the outcomes. Had we used concentrations, we would have been limited to assessing associations with only ∼5 HMOs. Another limitation was that we analyzed milk samples at 1 time point, so we were not able to capture the intraindividual variability (diurnal, day-to-day, or lactation stage) in these milk constituents. This could have caused us to miss associations with constituents that were transiently low or high at the time of collection. We did not measure the volume of breast milk consumed, preventing us from assessing how total infant intake of these breast milk constituents might be related to the outcomes, although others have reported that the associations of HMOs with infant growth were similar regardless of whether the exposure was defined as HMO concentration or total intake (18). Lastly, the number of nonsecretor women in our sample was relatively small, which limited our power to detect associations among nonsecretors. This could be the reason why we found more associations to be statistically significant in secretors than in nonsecretors.

In conclusion, we found the concentrations of particular bioactive breast milk proteins and abundance of HMOs in Malawian women to be significantly associated with infant growth and/or development, particularly in secretors. These results can help us to understand the extent to which breast milk composition contributes to optimal growth and development.

## Supplementary Material

nqaa272_Supplemental_File

## Notes

This publication is based on research funded in part by grants from the Bill & Melinda Gates Foundation to the University of California, Davis, and to Washington University, St Louis.

Supplemental Tables 1 and 2 and Supplemental Figure 1 are available from the “Supplementary data” link in the online posting of the article and from the same link in the online table of contents at https://academic.oup.com/ajcn/.

Abbreviations used: BH, Benjamini–Hochberg; BMMI, Breast Milk, Microbiome, and Immunity (project); DFLNH, difucosyllacto-*N*-hexaose; Hb, hemoglobin; HCZ, head circumference *z*-score; HHAZ, household asset *z*-score; HMO, human milk oligosaccharide; IFLNH, fucosyl-*para*-lacto-*N*-hexaose; LAZ, length-for-age *z*-score; LDFT, lactodifucotetraose; LNFP, lacto-*N*-fucopentaose; LNnH, lacto-*N*-neohexaose; LNnT, lacto-*N*-neotetraose; LNS, lipid-based nutrient supplement; LNT, lacto-*N*-tetraose; LST, sialyllacto-*N*-tetraose; WAZ, weight-for-age *z*-score; WLZ, weight-for-length *z*-score; ΔHCZ, change in head circumference z-score; ΔLAZ, change in length-for-age *z*-score; ΔWLZ, change in weight-for-length *z*-score; 2′FL, 2′-fucosyllactose.
