# Supplementary material for: Associations of human milk oligosaccharides and bioactive proteins with infant growth and development among Malawian mother-infant dyads
Source: Am J Clin Nutr. 2020 Oct 23;113(1):209–20. doi: 10.1093/ajcn/nqaa272 (PMC7779225; doi:10.1093/ajcn/nqaa272)
Supplement: nqaa272_Supplemental_File [file nqaa272_supplemental_file.pdf]

Associations of human milk oligosaccharides and bioactive proteins with infant growth and development among Malawian mother-infant dyads.

Josh M Jorgensen, et al.

Online Supplementary Material

Supplementary Table 1. Spearman's correlation coefficients for associations of bioactive proteins with human milk oligosaccharides among Malawian women at 6 mo postpartum. DFLNH, difucosyllacto-N-hexaose; DFLNnO, difucosyllacto-N-neooctaose; DFS-LNnH, difucosylmonosialyllacto-N-neohexaose; F-LST, fucosyl-sialyllacto-N-tetraose; IFLNH, fucosyl-para-lacto-N-hexaose; LDFT, lactodifucotetraose; LNDFH, lacto-N-difucohexaose; LNFP, lacto-N-fucopentaose; LNH, lacto-N-hexaose; LNnH, lacto-N-neothexaose; LNnT, lacto-N-neotetraose; LNT, lacto-N-tetraose; LST, sialyllacto-N-tetraose; MFpLNH IV, fucosyl-para-lacto-N-hexaose; p-LNH, para-lacto-N-hexaose; S-LNnH, sialyllacto-N-neohexaose; TFLNH, trifucosyllacto-N-hexaose; 2'FL, 2'-fucosyllactose; 3'FL, 3'-fucosyllactose; 3'SL, 3'-sialyllactose; 6'SL, 6'-sialyllactose. HMOs identified by numbers have not been previously named. Their composition is given as Hexose_N-acetylhexoseamine (HexNAc)_Fucose_N-Acetylneuraminic acid (sialic acid). For example, 5311 has 5 Hexoses, 3 HexNAc, 1 fucose, and 1 sialic acid. Thus, those with a non-zero number in the last position are sialylated; those with a non-zero number in the 3rd position are fucosylated.

		Absolute abundance HMOs	%Fucosylated HMOs	%Sialylated HMOs	%Fucosylated or sialylated HMOs	%Non-fucosylated neutral HMOs	%3FL	%2FL	%LDFT	%LNT
**Antitrypsin**	**Coefficient**	0.09013	0.04837	0.12542	0.13905	-0.05011	0.01436	0.03648	-0.07845	-0.08442
	**p-value**	0.0227	0.2221	0.0015	0.0004	0.2058	0.7171	0.3573	0.0474	0.0329
**IgA**	**Coefficient**	0.06158	0.05146	0.16764	0.09986	-0.07978	0.06306	-0.06645	0.01217	-0.00117
	**p-value**	0.12	0.1939	<.0001	0.0115	0.0438	0.1113	0.0933	0.7589	0.9765
**Lactalbumin**	**Coefficient**	0.04537	-0.00662	0.12868	0.18484	0.01084	0.03771	0.01469	-0.16295	0.07825
	**p-value**	0.2522	0.8673	0.0011	<.0001	0.7844	0.3413	0.7108	<.0001	0.048
**Lactoferrin**	**Coefficient**	0.20304	0.08045	0.05055	0.06184	-0.07269	-0.03986	0.02572	-0.0255	-0.03425
	**p-value**	<.0001	0.0421	0.2019	0.1184	0.0663	0.3144	0.5163	0.5199	0.3873
**Lysozyme**	**Coefficient**	0.03992	-0.01906	0.10655	0.07536	-0.00403	-0.02997	-0.12118	-0.03826	0.04191
	**p-value**	0.3136	0.6305	0.007	0.0569	0.9191	0.4494	0.0022	0.3342	0.2902
**Osteopontin**	**Coefficient**	0.06398	-0.00183	0.05333	0.04086	0.00507	-0.09073	-0.00681	-0.18391	-0.02355
	**p-value**	0.1061	0.9631	0.1782	0.3024	0.8982	0.0218	0.8636	<.0001	0.5524

		%LNnT	%LNT + LNnT	%LNFP II	%LNFP I + III	%LNH	%LNnH	%p-LNH	%MFpLNH IV	%4120a	%MFLNH III + I
**Antitrypsin**	**Coefficient**	-0.06466	-0.10733	-0.07207	-0.04497	0.02334	0.03022	0.02444	-0.08557	-0.162	-0.08964
	**p-value**	0.1024	0.0066	0.0686	0.2564	0.556	0.4457	0.5374	0.0306	<.0001	0.0234
**IgA**	**Coefficient**	-0.12259	-0.04513	-0.00572	0.00106	-0.12993	-0.13935	-0.0852	-0.11612	-0.00433	-0.10387
	**p-value**	0.0019	0.2546	0.8853	0.9786	0.001	0.0004	0.0313	0.0033	0.9129	0.0086
**Lactalbumin**	**Coefficient**	-0.18791	-0.02639	-0.04737	-0.08873	0.05044	-0.05519	-0.03772	-0.14212	-0.20181	0.01505
	**p-value**	<.0001	0.5055	0.2318	0.0249	0.2029	0.1635	0.3411	0.0003	<.0001	0.7042
**Lactoferrin**	**Coefficient**	-0.13712	-0.10047	0.0186	0.10074	-0.03215	-0.06299	0.07442	-0.19905	-0.03502	-0.14209
	**p-value**	0.0005	0.011	0.6388	0.0108	0.4173	0.1117	0.0601	<.0001	0.3768	0.0003
**Lysozyme**	**Coefficient**	-0.07494	-0.01048	0.00372	-0.02628	0.04331	-0.06325	-0.11697	-0.01659	0.02644	0.05687
	**p-value**	0.0583	0.7914	0.9253	0.5073	0.2743	0.1102	0.0031	0.6756	0.5047	0.151
**Osteopontin**	**Coefficient**	-0.1041	-0.08093	-0.09178	-0.0286	0.12793	0.01881	0.07697	-0.0352	-0.10565	-0.01274
	**p-value**	0.0084	0.0409	0.0203	0.4704	0.0012	0.6352	0.0518	0.3743	0.0075	0.7478

		%IFLNH III	%IFLNH I	%DFpLNH II	%DFLNHb	%DFLNHa	%DFLNHc	%TFLNH	%4320a	%5130a	%5130b
**Antitrypsin**	**Coefficient**	-0.03713	-0.22323	-0.09591	-0.11146	0.02065	-0.22692	-0.0472	-0.21693	0.00561	-0.1489
	**p-value**	0.3487	<.0001	0.0153	0.0048	0.6024	<.0001	0.2335	<.0001	0.8874	0.0002
**IgA**	**Coefficient**	-0.13754	-0.05397	-0.03175	-0.04484	-0.04033	-0.0194	0.02558	0.0341	-0.07927	-0.08119
	**p-value**	0.0005	0.173	0.423	0.2577	0.3087	0.6245	0.5187	0.3895	0.0452	0.0402
**Lactalbumin**	**Coefficient**	-0.16842	-0.33905	-0.19342	-0.11599	-0.01243	-0.32062	-0.10934	-0.34059	0.11168	-0.23073
	**p-value**	<.0001	<.0001	<.0001	0.0033	0.7538	<.0001	0.0057	<.0001	0.0047	<.0001
**Lactoferrin**	**Coefficient**	-0.24473	-0.05492	-0.1466	-0.18269	0.06438	-0.04399	0.07516	0.00323	-0.03618	-0.11945
	**p-value**	<.0001	0.1655	0.0002	<.0001	0.104	0.2669	0.0576	0.935	0.3612	0.0025
**Lysozyme**	**Coefficient**	0.0139	-0.1224	0.01088	0.03257	-0.0256	-0.19867	0.00476	-0.18537	0.10016	-0.12389
	**p-value**	0.7258	0.0019	0.7837	0.4111	0.5182	<.0001	0.9044	<.0001	0.0113	0.0017
**Osteopontin**	**Coefficient**	-0.07979	-0.2081	-0.09771	-0.07439	0.03039	-0.23983	-0.08254	-0.19597	0.08332	-0.20225
	**p-value**	0.0438	<.0001	0.0135	0.0602	0.4431	<.0001	0.037	<.0001	0.0352	<.0001

		%F-LNO	%5130c	%DFLNO I	%DFLNnO II	%5230a	%DFLNnO I + DFLNO II	%5230 + DLFNO1	%5230b	%5330a	%4240a
**Antitrypsin**	**Coefficient**	-0.05805	-0.26511	0.00098	-0.07974	-0.0026	0.16716	0.14299	0.15104	0.32447	0.32427
	**p-value**	0.1427	<.0001	0.9802	0.0439	0.9477	<.0001	0.0003	0.0004	<.0001	<.0001
**IgA**	**Coefficient**	-0.14552	-0.00868	-0.11661	0.0421	0.11412	-0.12638	-0.02344	0.04811	0.03682	0.02186
	**p-value**	0.0002	0.8267	0.0032	0.2879	0.0039	0.0014	0.5542	0.2596	0.3527	0.5812
**Lactalbumin**	**Coefficient**	-0.15544	-0.32629	0.14807	-0.14192	-0.14305	0.17141	0.09864	0.09517	0.40756	0.38803
	**p-value**	<.0001	<.0001	0.0002	0.0003	0.0003	<.0001	0.0126	0.0255	<.0001	<.0001
**Lactoferrin**	**Coefficient**	-0.15718	-0.04974	-0.0945	0.03218	0.01227	0.11247	0.11041	-0.08201	0.23421	0.22715
	**p-value**	<.0001	0.2092	0.0169	0.4167	0.757	0.0044	0.0052	0.0544	<.0001	<.0001
**Lysozyme**	**Coefficient**	0.02578	-0.32133	0.05458	0.09599	0.34301	0.00325	0.24666	0.20771	0.00474	0.00358
	**p-value**	0.5154	<.0001	0.1682	0.0152	<.0001	0.9346	<.0001	<.0001	0.9047	0.928
**Osteopontin**	**Coefficient**	-0.07911	-0.31812	-0.04039	-0.06	0.13005	0.146	0.20733	-0.00187	0.33575	0.34673
	**p-value**	0.0456	<.0001	0.308	0.1298	0.001	0.0002	<.0001	0.9651	<.0001	<.0001

		%5300a	%6400a	%6400b	%6SL	%3SL	%LSTc	%LSTb	%LSTa	%F-LSTc	%S-LNH
**Antitrypsin**	**Coefficient**	0.09877	0.32239	0.14199	-0.08391	-0.07009	0.02717	-0.0121	0.00206	0.06551	-0.01549
	**p-value**	0.0125	<.0001	0.0003	0.034	0.0766	0.4929	0.7601	0.9586	0.098	0.6959
**IgA**	**Coefficient**	-0.15458	0.03714	-0.01069	0.12969	0.11168	0.10691	0.05258	-0.12589	-0.06405	0.00845
	**p-value**	<.0001	0.3486	0.7875	0.001	0.0047	0.0068	0.1844	0.0014	0.1058	0.8312
**Lactalbumin**	**Coefficient**	0.05102	0.37814	0.12655	-0.12918	-0.17315	-0.01887	0.04	0.07811	0.10875	0.03278
	**p-value**	0.1978	<.0001	0.0013	0.0011	<.0001	0.6339	0.3127	0.0484	0.0059	0.4081
**Lactoferrin**	**Coefficient**	-0.02964	0.22985	0.10855	0.10599	-0.17697	0.06299	-0.03365	-0.11254	0.00647	0.01584
	**p-value**	0.4546	<.0001	0.006	0.0073	<.0001	0.1116	0.3958	0.0044	0.8703	0.6893
**Lysozyme**	**Coefficient**	-0.06538	0.00578	-0.01657	-0.06583	0.1154	-0.06202	0.07621	0.00803	-0.20735	0.0015
	**p-value**	0.0987	0.8841	0.6759	0.0964	0.0035	0.1173	0.0542	0.8395	<.0001	0.9699
**Osteopontin**	**Coefficient**	0.10038	0.34548	0.13769	0.08988	-0.0603	-0.09394	-0.03541	0.06637	-0.05606	-0.00301
	**p-value**	0.0111	<.0001	0.0005	0.0231	0.1279	0.0175	0.3715	0.0937	0.1569	0.9394

		%4021+ S-LNnH II	%DFS-LNnH	%5311a	%4211a	%4211b	%4211c	%4100a	%4100b	Antitrypsin	IgA6
Antitrypsin	Coefficient	0.09493	0.18724	0.32071	0.18159	0.18239	-0.01732	0.01793	0.06556		0.08706
	p-value	0.0164	<.0001	<.0001	<.0001	<.0001	0.685	0.6745	0.1243		0.0278
IgA	Coefficient	-0.00851	-0.11254	0.01198	-0.12082	-0.11494	0.0965	-0.11473	0.09905	0.08706	
	p-value	0.83	0.0082	0.7625	0.0045	0.0069	0.0235	0.007	0.02	0.0278	
Lactalbumin	Coefficient	0.02244	0.3197	0.39559	0.32389	0.32215	-0.03271	0.01001	0.15569	0.34856	0.05303
	p-value	0.5712	<.0001	<.0001	<.0001	<.0001	0.4435	0.8146	0.0002	<.0001	0.1806
Lactoferrin	Coefficient	0.10624	0.12329	0.22313	0.11852	0.11742	-0.0785	-0.16708	0.05579	0.31261	0.50298
	p-value	0.0072	0.0037	<.0001	0.0053	0.0058	0.0656	<.0001	0.191	<.0001	<.0001
Lysozyme	Coefficient	-0.05718	0.00143	-0.00602	0.00294	0.00125	-0.02792	-0.05073	0.09188	0.24488	0.16822
	p-value	0.1488	0.9733	0.8793	0.9451	0.9767	0.513	0.2345	0.031	<.0001	<.0001
Osteopontin	Coefficient	0.06645	0.2662	0.34675	0.26555	0.26715	-0.1086	-0.09432	-0.05455	0.37555	0.2336
	p-value	0.0933	<.0001	<.0001	<.0001	<.0001	0.0107	0.0268	0.201	<.0001	<.0001

		Lactalbumin	Lactoferrin	Lysozyme	Osteopontin
Antitrypsin	Coefficient	0.34856	0.31261	0.24488	0.37555
	p-value	<.0001	<.0001	<.0001	<.0001
IgA	Coefficient	0.05303	0.50298	0.16822	0.2336
	p-value	0.1806	<.0001	<.0001	<.0001
Lactalbumin	Coefficient		0.16708	0.11339	0.29788
	p-value		<.0001	0.0041	<.0001
Lactoferrin	Coefficient	0.16708		0.30991	0.54752
	p-value	<.0001		<.0001	<.0001
Lysozyme	Coefficient	0.11339	0.30991		0.54986
	p-value	0.0041	<.0001		<.0001
Osteopontin	Coefficient	0.29788	0.54752	0.54986	
	p-value	<.0001	<.0001	<.0001	

Supplementary Table 2. Mean (SD) or prevalence (%) of Malawian infant growth and development variables at the 1st and 5th quintile of human milk oligosaccharide relative abundance for the exploratory analyses that were significant upon linear regression modeling.

sample	HMO	quintile	ΔLAZ	ΔWAZ	ΔWLZ	ΔHCZ	Standing	Walking	Motor skills	Language	PSED	A-B correct
Full sample	%4320a	1	-0.3 (0.7)									
Full sample	%4320a	5	-0.1 (0.8)									
Full sample	%5130c	1								30.5 (23.6)		
Full sample	%5130c	5								25.3 (23.1)		
Full sample	%5230b	1		-0.3 (0.7)	-0.5 (0.8)							
Full sample	%5230b	5		-0.1 (0.6)	-0.2 (0.9)							
Full sample	%5330a	1				-0.4 (0.6)						
Full sample	%5330a	5				-0.4 (0.5)						
Full sample	%6400a	1		-0.2 (0.5)								
Full sample	%6400a	5		-0.1 (0.5)								
Full sample	%6400b	1		-0.1 (0.5)								
Full sample	%6400b	5		-0.2 (0.5)								
Full sample	%5311a	1	-0.4 (0.7)	-0.2 (0.5)								
Full sample	%5311a	5	-0.1 (0.7)	-0.1 (0.5)								
Secretors	%Fucosylated and sialylated HMOs	1								26.4 (22.6)		
Secretors	%Fucosylated and sialylated HMOs	5								29.7 (22.3)		
Secretors	%Non-fucosylated neutral HMOs	1								31.9 (23.4)		
Secretors	%Non-fucosylated neutral HMOs	5								31.5 (24.6)		
Secretors	%LDFT	1	-0.2 (0.7)		-0.5 (0.8)					32.7 (24.2)		
Secretors	%LDFT	5	-0.4 (0.6)		-0.2 (0.9)					34.9 (23.5)		
Secretors	%LNT	1		-0.2 (0.6)	-0.3 (0.8)	-0.4 (0.5)						
Secretors	%LNT	5		-0.2 (0.8)	-0.5 (0.9)	-0.5 (0.7)						
Secretors	%LNnT	1						48.3		35.7 (23.4)		
Secretors	%LNnT	5						60.9		27.4 (22.9)		
Secretors	%LNT + LNnT	1			-0.3 (0.8)					31.0 (22.4)		
Secretors	%LNT + LNnT	5			-0.5 (0.9)					33.9 (25.2)		
Secretors	%LNFP I + III	1			-0.3 (0.7)					36.5 (25.5)		
Secretors	%LNFP I + III	5			-0.5 (0.8)					24.9 (22.4)		

sample	HMO	quintile	ΔLAZ	ΔWAZ	ΔWLZ	ΔHCZ	Standing	Walking	Motor skills	Language	PSED	A-B correct
Secretors	%LNH	1								29.6 (22.6)		
Secretors	%LNH	5								27.7 (25.0)		
Secretors	%LNnH	1								32.3 (22.2)		
Secretors	%LNnH	5								28.8 (23.3)		
Secretors	%p-LNH	1								32.2 (24.1)		
Secretors	%p-LNH	5								30.5 (24.9)		
Secretors	%MFpLNH IV	1								28.4 (22.4)		
Secretors	%MFpLNH IV	5								32.5 (25.5)		
Secretors	%IFLNH I	1			-0.2 (0.8)					32.4 (23.9)		
Secretors	%IFLNH I	5			-0.5 (0.9)					23.8 (20.6)		
Secretors	%DFLNHa	1										6.1 (2.7)
Secretors	%DFLNHa	5										6.6 (2.4)
Secretors	%DFLNHc	1	-0.4 (0.8)		-0.1 (0.8)							
Secretors	%DFLNHc	5	-0.2 (0.8)		-0.5 (0.8)							
Secretors	%5130a	1						59.8				
Secretors	%5130a	5						54.4				
Secretors	%5130b	1	-0.3 (0.7)									
Secretors	%5130b	5	-0.2 (0.8)									
Secretors	%5130c	1								30.5 (23.6)		
Secretors	%5130c	5								25.3 (23.1)		
Secretors	%DFLNnO II	1							39.1 (4.0)			
Secretors	%DFLNnO II	5							38.0 (4.1)			
Secretors	%4240a	1	-0.4 (0.7)									
Secretors	%4240a	5	-0.2 (0.6)									
Secretors	%5300a	1								32.0 (22.9)		
Secretors	%5300a	5								27.4 (22.9)		
Secretors	%6400a	1						50.8				
Secretors	%6400a	5						46.1				
Secretors	%6SL	1				-0.4 (0.6)		50.4				
Secretors	%6SL	5				-0.4 (0.7)		43.5				
Secretors	%LSTc	1									22.4 (5.6)	
Secretors	%LSTc	5									21.3 (5.3)	

sample	HMO	quintile	ΔLAZ	ΔWAZ	ΔWLZ	ΔHCZ	Standing	Walking	Motor skills	Language	PSED	A-B correct
Secretors	%LSTa	1	-0.3 (0.7)		-0.3 (0.9)							
Secretors	%LSTa	5	-0.2 (0.7)		-0.5 (0.8)							
Secretors	%F-LSTc	1							38.2 (4.5)			
Secretors	%F-LSTc	5							39.1 (3.4)			
Secretors	%v4021 + S-LNnH II	1									22.3 (5.4)	
Secretors	%v4021 + S-LNnH II	5									21.3 (5.2)	
Secretors	%DFS-LNnH	1					83.2			24.2 (20.9)		
Secretors	%DFS-LNnH	5					83.5			37.5 (26.6)		
Secretors	%4211c	1								28.5 (24.4)		
Secretors	%4211c	5								31.4 (23.7)		
Non-secretors	%LNFP II	1			-0.4 (0.8)				38.4 (4.1)			
Non-secretors	%LNFP II	5			-0.6 (0.8)				38.0 (5.0)			
Non-secretors	%DFLNHb	1									22.0 (5.7)	
Non-secretors	%DFLNHb	5									21.3 (5.6)	
Non-secretors	%LSTb	1										6.5 (2.6)
Non-secretors	%LSTb	5										6.5 (2.6)
Non-secretors	%DFLNHc	1							38.5 (3.7)			
Non-secretors	%DFLNHc	5							38.2 (4.4)			
Non-secretors	%TFLNH	1							38.2 (4.6)	31.7 (24.8)		
Non-secretors	%TFLNH	5							38.1 (4.3)	31.2 (22.9)		

sample	HMO	quintile	ΔLAZ	ΔWAZ	ΔWLZ	ΔHCZ	Standing	Walking	Motor skills	Language	PSED	A-B correct
Non-secretors	%4320a	1							38.8 (3.4)			
Non-secretors	%4320a	5							38.4 (4.0)			
Non-secretors	%5130a	1							38.5 (4.1)			
Non-secretors	%5130a	5							38.8 (4.5)			
Non-secretors	%5130b	1							38.1 (4.2)			
Non-secretors	%5130b	5							39.0 (4.0)			
Non-secretors	%4211b	1								24.2 (20.9)		
Non-secretors	%4211b	5								37.5 (26.6)		
Non-secretors	%4120a	1			-0.3 (0.8)							
Non-secretors	%4120a	5			-0.4 (0.9)							
Non-secretors	%IFLNH III	1										6.3 (2.5)
Non-secretors	%IFLNH III	5										6.4 (2.5)
Non-secretors	%IFLNH I	1							38.8 (3.6)			
Non-secretors	%IFLNH I	5							37.5 (4.9)			

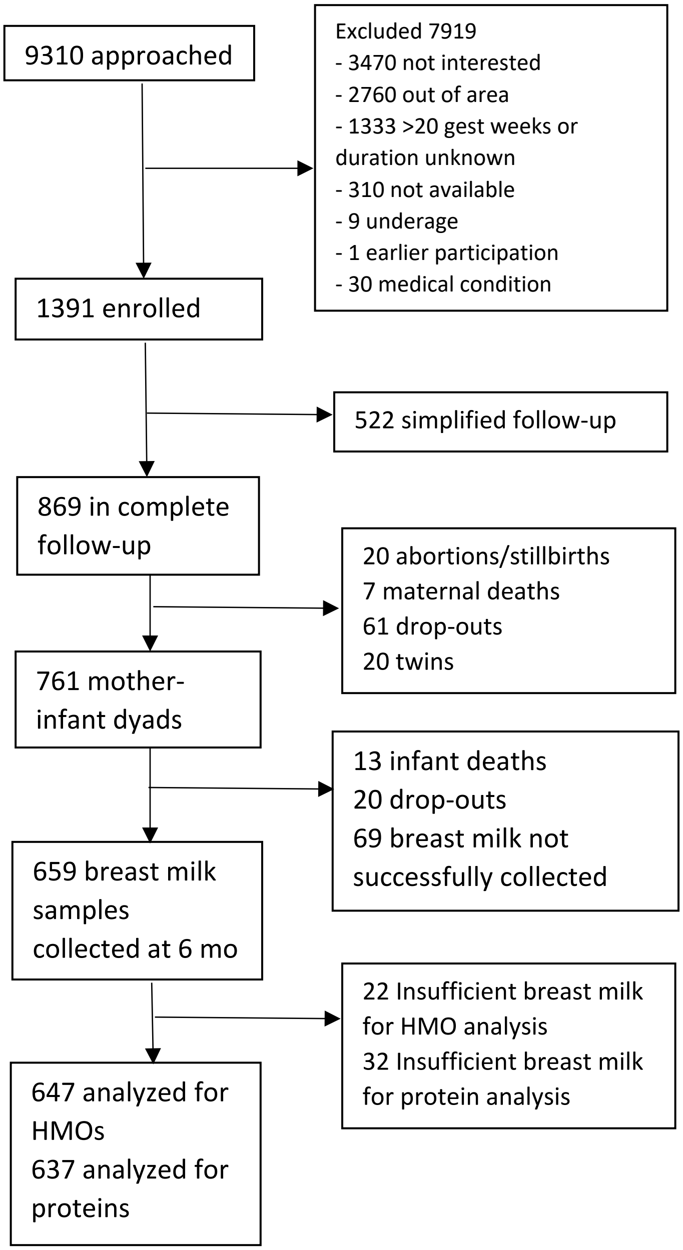

Supplementary Figure 1. Flow chart of participant enrollment and sample collection of Malawian women-infant dyads who participated in the International Lipid-Based Nutrient Supplement (iLiNS) Project and were included in the analysis of associations of Human Milk Oligosaccharides (HMOs) and bioactive breast milk proteins with infant growth and development.
